# Trajectories of Antiretroviral Therapy Adherence and Virologic Failure in Women with HIV in the United States

**DOI:** 10.1097/QAI.0000000000003174

**Published:** 2023-02-13

**Authors:** Abubaker Ibrahim Elbur, Musie Ghebremichael, Deborah Konkle-Parker, Deborah L Jones, Shelby Collins, Adaora A. Adimora, Michael F. Schneider, Mardge H. Cohen, Bani Tamraz, Michael Plankey, Tracey Wilson, Adebola Adedimeji, Jessica Haberer, Denise L. Jacobson

**Affiliations:** 1. Ph.D., Center for Global Health, Massachusetts General Hospital, Boston, MA, USA; 2. Musie Ghebremichael (Ph.D.), The Ragon Institute of MGH, MIT, and Harvard, Cambridge, MA, USA; 3. Ph.D., FNP, Schools of Nursing, Medicine and Population Health, University of Mississippi Medical Center, Jackson, MS, USA; 4. Ph.D.; Department of Psychiatry & Behavioral Sciences, University of Miami Miller School of Medicine, Miami, FL, USA; 5. MSN; NP-C; Emory University School of Medicine, Division of Infectious Disease, Atlanta, GA, USA; 6. MD, MPH, Department of Medicine, University of North Carolina at Chapel Hill, Chapel Hill, NC, USA; 7. MS; Department of Epidemiology, Johns Hopkins Bloomberg School of Public Health, Baltimore, USA; 8. MD; Department of Medicine, Stroger Hospital of Cook County, Chicago IL, USA; 9. Pharm. D, Ph.D; University of California, San Francisco, School of Pharmacy, San Francisco, CA, USA; 10. Ph.D.; Georgetown University Medical Center, Department of Medicine, Division of General Internal Medicine, Washington, DC, USA; 11. Ph.D.; School of Public Health, SUNY Downstate Health Sciences University, Brooklyn, NY; 12. Ph.D., MS, MPH, MBA, Dept of Epidemiology and Population Health, Albert Einstein College of Medicine, Bronx, NY, USA; 13. MD; MPH; Center for Global Health, Massachusetts General Hospital, Boston, MA, USA; 14. Ph.D., MPH; Center for Biostatistics in AIDS Research, Department of Epidemiology, Harvard T. H. Chan School of Public Health, Boston, MA, USA

**Keywords:** Medication Adherence, Antiretroviral therapy, Group-based trajectory modeling, Virologic failure, Women

## Abstract

**Background:**

Women with HIV (WHIV) in the United States face many challenges with adherence to antiretroviral therapy (ART), and suboptimal adherence often leads to virologic failure. This study aimed to determine the association between ART adherence trajectories and the risk of virologic failure.

**Methods:**

We included WHIV (aged ≥ 18) enrolled in the Women’s Interagency HIV Study in the US from April 2014 to September 2019 who had at least two consecutive measurements of HIV RNA and ≥3 measurements of adherence. Group-based trajectory modeling (GBTM) was used to identify adherence trajectories. Cox proportional hazard ratios were used to measure the association.

**Main outcome measure:**

Virologic failure, was defined as HIV RNA ≥200 copies/mL at two consecutive visits.

**Results:**

We included 1,437 WHIV (median age 49 years). Of all women, 173 (12.0%) experienced virologic failure. Four adherence trajectories were identified, namely ‘consistently high’ (26.3%), ‘moderate increasing’ (9.5%), ‘moderate decreasing’ (30.6%), and ‘consistently low’ (33.5%). Women in the consistently low adherence group consumed alcohol and experienced depression more than other groups. Compared to the ‘consistently high’ trajectory, the risk of virologic failure was higher among women with ‘consistently low’ (adjusted hazard ratio (aHR) 2.8; 95% CI: 1.6-4.9; P < 0.001) and ‘moderate decreasing’ adherence trajectories (aHR 1.8; 95% CI: 1.0-3.2; P =0.04), but it was similar to those with ‘moderate increasing’ adherence trajectory (aHR 1.0; 95% CI: 0.4-2.5; P = 0.94).

**Conclusion:**

Adherence to ART remains a challenge among WHIV. Multilevel behavioral interventions to address poor adherence, alcohol consumption, and depression are needed.

## Introduction

In 2019, women accounted for 18% of the 34,800 new HIV cases diagnosed in the United States [1], while The Centers for Disease Control and Prevention (CDC) estimated that by the end of 2019, women with HIV (WHIV) constituted approximately 22% of all people living with HIV (PWHIV) in the United States [2].

HIV antiretroviral therapy (ART) maintains health and well-being and reduces HIV transmission, which can be achieved through viral suppression [3]. Viral suppression among women is high, for example, Kassaye et al [4] characterized the long-term HIV viral suppression among nearly two thousand women enrolled in the Women’s Interagency HIV Study (WIHS); between 2015 and 2017, only 70% of the women demonstrated sustained viral suppression.

Suboptimal adherence to ART is also high among women as compared to men, for example, an analysis of a large sample of PWHIV data in the US obtained from the Integrated Dataverse (IDV) between January 2015 and September 2017 (n=169,545; 27% female) revealed that female gender was a strong predictor of poor adherence and greater prevalence of drug resistance [5]. Turner *et al* [6] similarly evaluated the relationship between gender and adherence to ART measured by the proportion of days covered (PDC) using pharmacy data among people who use drugs living with HIV in the US (1,827 female and 3,216 male); women were found to be significantly less adherent than men (18% vs 25%, *P* < 0.001). Multiple factors noted in the literature that are associated with a decreased level of adherence among women include alcohol dependence, depression, anxiety, internalized stigma, childcare, other competing life demands, and a history of physical and sexual abuse [7,8].

Variations in patterns of adherence over time can influence the likelihood of maintaining viral suppression. Most studies have examined the within-subject patterns of adherence over time [9. 10]. Static measures, such as the medication possession ratio, are insufficient to capture the dynamic nature of long-term adherence behavior [11]. Group-based trajectory modeling (GBTM) is a novel methodological data-driven approach used for analyzing developmental trajectories (i.e., the evolution of an outcome over age or time) [12] and can be used to categorize trajectories of adherence, rather than simply dichotomizing participants as adherent versus nonadherent. GBTM has increasingly been utilized to study individuals’ adherence to treatment across different disease states [11]. Identifying trajectories may be advantageous in the customization of targeted interventions to increase adherence and improve treatment outcomes, as interventions focusing on patients at risk of poor adherence rather than on all patients can produce better outcomes [13].

Few studies to date have examined the relationship between medication adherence trajectories and health care events and/or treatment outcomes [14,15, 16]. We hypothesized that GBTM analysis will delineate different latent trajectories of adherence to ART, and WIHS women who follow a low adherence trajectory over time are at an increased risk of experiencing virologic failure compared to those with moderate or high adherence patterns.

## Methods

### Study design

We conducted a retrospective analysis of longitudinal data among WHIV enrolled in WIHS, which was the oldest and largest prospective cohort of WHIV or women living without HIV (assigned the female sex at birth) in the US [17,18]; the WIHS is now combined with the Multicenter AIDS Cohort Study (MACS) to form the MACS/WIHS Combined Cohort Study (MWCCS). WIHS recruited women during four waves (1994-95, 2001-2002, 2011-2012, and 2013-2015) from the Bronx and Brooklyn, New York; Washington, DC; Los Angeles and San Francisco, California; and Chicago, Illinois. More participants were enrolled during the fourth wave from other research sites in Atlanta, Georgia; Chapel Hill, North Carolina; Miami, Florida; Birmingham, Alabama; and Jackson, Mississippi [19]. The study was approved by the Institutional Review Board at each study site’s institution.

### WIHS data

At each semi-annual study visit, data collection included clinical exams, blood samples, and interviewer-administered questionnaires to collect basic sociodemographic data, substance use, non-HIV and HIV medication use, HIV viral load, and CD4 count. At each semi-annual visit, data were collected on socio-demographic characteristics, including age (years), race and ethnicity (non-Hispanic white, non-Hispanic African American, and Hispanic of any race), educational level (below secondary, completed secondary, some college/completed college), household income (here categorized as <$24,000 vs. ≥$24,000), employment status (employed vs. unemployed), estimated or self-reported time since diagnosis with HIV (years), smoking status (never smoker, current smoker, former smoker), alcohol intake (abstainer, >0-7 drinks/week, >7-12 drinks/week, >12 drinks/week), substance use (marijuana or hash, crack, cocaine, heroin, illicit methadone, methamphetamines, amphetamines, narcotics, hallucinogens, other drugs), and depression status (measured by the Center for Epidemiological Studies Depression (CES-D) Scale with a score of ≥16 indicating the presence of depressive symptoms and <16 indicating no depression symptoms [20]. The type of ART regimen used at the last visit was also recorded (here categorized as integrase strand transfer inhibitors [INSTI] without protease inhibitors [PI] and non-nucleoside reverse transcriptase inhibitor [NNRTI], NNRTI without PIs, PIs alone, no therapy, others).

Self-reported data on adherence to ART over the past six months is also collected at each visit. Response options include: “100% of the time”, “95-99% of the time”, “75-94% of the time”, “<75% of the time,” and “I have not taken any of my prescribed medications.”

### Study participants

The sample population for the current analysis consisted of WHIV enrolled in WIHS from April 2014 to September 2019. Our analysis included all WHIV aged ≥18 years, who were taking ART and had at least two consecutive measurements of HIV RNA. In addition, we excluded women with fewer than three measurements of adherence because of the minimum requirements for fitting trajectory models. The index visit was defined as the first visit between the above dates.

### Data analysis

#### Main outcome measure

The main outcome measure was virologic failure, defined as HIV RNA ≥200 copies/mL at two consecutive visits [21].

#### Primary Exposure

Adherence to antiretroviral therapy trajectories as categorized by GBTM.

### Statistical Analysis

#### Group-based trajectory modeling

We used GBTM to identify latent adherence groups using a censored normal finite mixture model [22]. The analysis process involved two steps. First, we started by sequentially fitting several models to identify the appropriate number of trajectory groups. The second step involved the identification of trajectory shapes considering constant, linear, quadratic, and cubic specifications, together with visual inspections. Model fit was determined by considering a combination of criteria, namely 1) Bayesian Information Criteria (BIC) with smaller values indicating better model fit, 2) the mean posterior probability of membership within each group (entropy) with values >0.70 generally indicating acceptable classification, 3) the smallest group with at least 5% of the sample, 4) a tight confidence interval around estimated group membership probabilities and statistically significant groups (*P*<0.05), and 5) parsimony in the model with few classes and parameters probabilities [12]. In addition to the statistical steps, the model selection process was based on subject matter knowledge about the patterns of adherence to medications and the interpretability of the model. We assumed that the missingness was missing completely at random. With this approach, GBTM accommodates missingness by fitting the model using maximum likelihood estimation and generating asymptotically unbiased parameter estimates [12].

#### Descriptive statistics

Categorical variables were presented as numbers and percentages and continuous variables were summarized as median and interquartile ranges (IQR) within each adherence trajectory group. We used the Chi-square test to compare age, race, education, employment status, annual income, alcohol intake, smoking, presence of depression symptoms at baseline, type of regimen at the last visit, and an episode of detectable viremia during the entire study period between the identified trajectories.

#### Adherence trajectories and virologic failure

The association between adherence trajectory groups (defined above) and time to virologic failure was assessed using the Kaplan-Meier method and a log-rank test was used to test for differences between the four adherence trajectory groups. Univariate and multivariate Cox’s proportional hazards (PH) models were used to estimate the hazard ratio (HR) as the measure of association between adherence trajectory group and virologic failure. The 95% confidence interval (CI) was used as a measure of precision. Based on the literature and expert knowledge, we selected as potential confounders, variables that may have a direct effect on viremia not mediated through adherence. Specifically, we included the type of ART regimen at the last visit and duration of prior viral suppression (estimated from episodes of detectable viremia), which may influence the threshold for viral replication [23,24], as well as alcohol intake, smoking, and depression [25,26,27]. Detectable viremia was defined as any detectable HIV RNA above the limit of detection (≥20 copies/mL) [28]. Episodes of detectable viremia were classified as “never” if women presented with viremia below the limit of detection at all of their visits, “infrequent” if women presented with detectable viremia in ≤50% of their total visits, and “frequently” if the women presented with detectable viremia in >50% of their total visits [29]. Statistical analyses were performed using Stata version 16 (Stata Corp LLC, College Station, Texas) and Stata Plugin was used to estimate GBTM parameters.

## Results

### Participant characteristics

**Figure 1:**
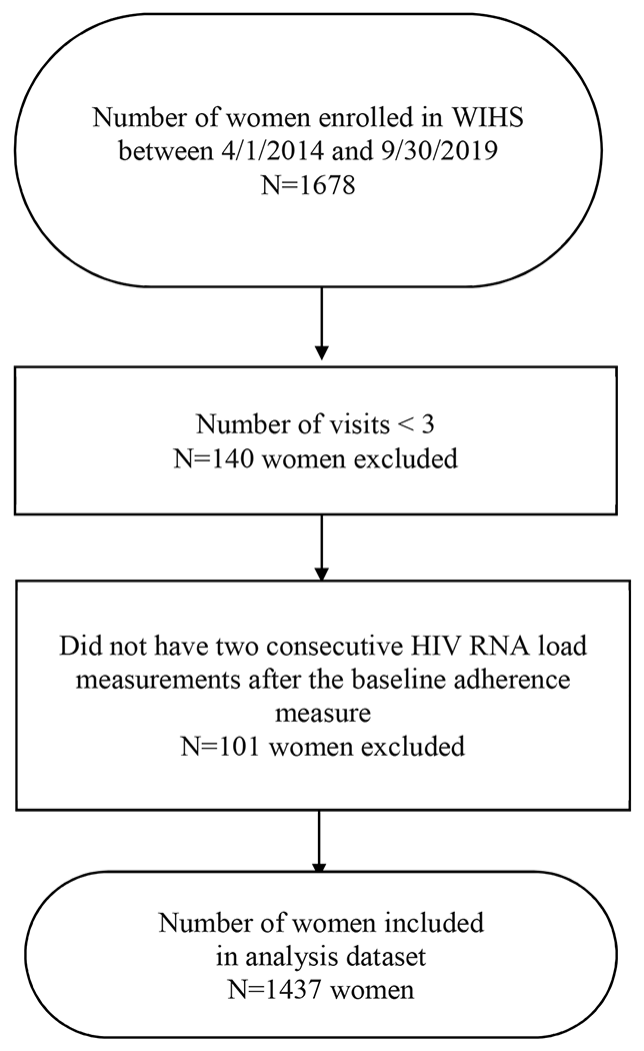
Sample selection process

Of the 1,678 women who were active in WIHS during the study period, 1,437 (86%) satisfied the inclusion criteria; they contributed a total of 13,056 participant-visits with a median follow-up time of approximately 53.6 (IQR 47-55) months (Figure 1). During the study period, HIV RNA was measured at 11,649 (89%) participant-visits and women self-reported adherence to ART at 12,389 (95%) participant-visits. Among all women in the study sample, the median age was 49 (IQR 42-54) years with the majority (1,059; 74%) self-identifying as African American. A total of 930 (65%) were unemployed, nearly three-quarters (76%) had annual income ≤$24,000, and 1,376 (96%) had medical insurance. Most women (1,122; 78%) were diagnosed with HIV at least 20 years before the baseline visit. See Table 1 for detailed participants’ characteristics overall and by adherence trajectory group.

### Trajectories of ART adherence and characteristics across groups

**Figure 2:**
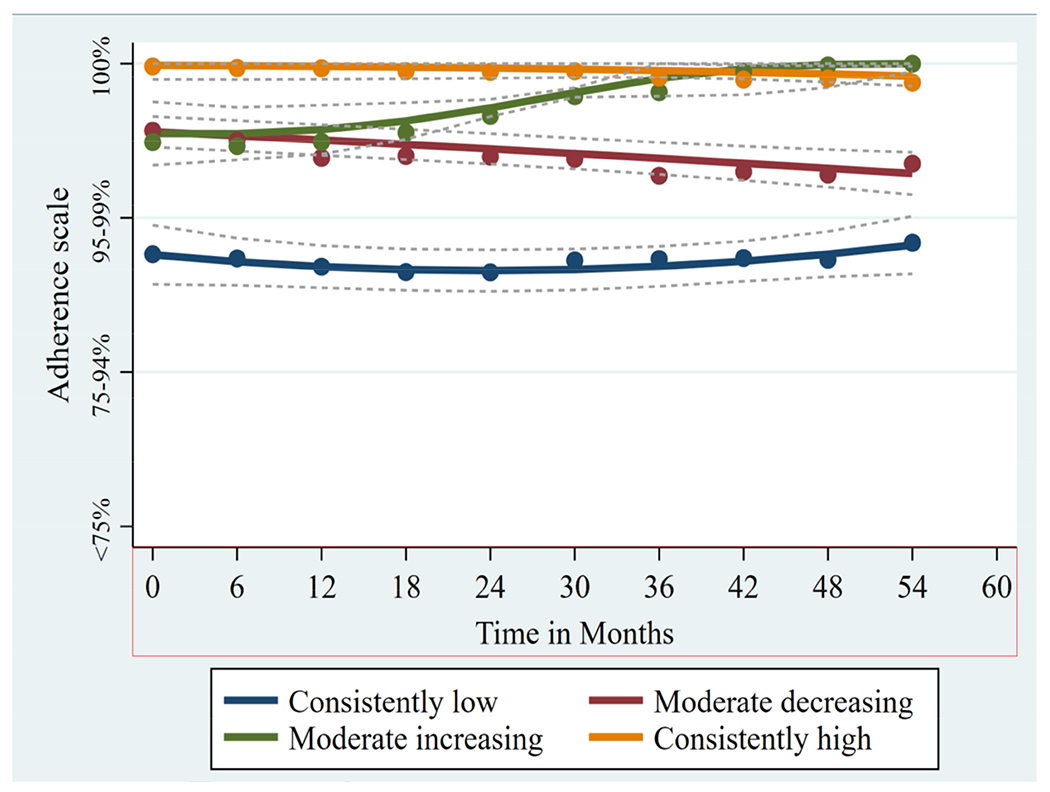
Trajectories of ART adherence

GBTM revealed four latent trajectories of adherence to ART, namely ‘consistently high’ (N=378, 26.3%), ‘moderate increasing’ (N=137, 9.5%), ‘moderate decreasing’ (N=440, 30.6%), and ‘consistently low’ (N=482, 33.5%), as depicted in Figure 2.

Race, employment status, and history of smoking status at the baseline visit were similar across groups (Table 1). A higher percentage of women in the consistently low adherence group used alcohol and experienced depression symptoms at the baseline visit compared to the other groups. In addition, women in the consistently low adherence group were likely to be taking INSTI and PI-based regimens, not taking therapy at the last visit, and frequently presented with detectable viremia during the whole study period.

Viral load was not detected in 161 (11.3%) participants in all study visits, of whom 61(16.1%) were in the consistently high group, 61 (13.9%) in the moderate decreasing, 10 (7.3%) in the moderate increasing, and 31 (6.4%) in the consistently low groups. Viral load was detectable in 5,030 (43.2%) visits. Of the women who experienced frequent episodes of viremia 109 (28.8%) were members of the consistently high adherence group, compared to 159 (36.1%) in the moderate decreasing, 54 (39.4%) in the moderate increasing, and 244 (50.6%) in the consistently low adherence group. Of the women who experienced infrequent viremia, 208 (55.0%) were in the consistently high adherence trajectory, compared to 73 (53.3%), 220 (50.0%), and 207 (49.3%) in the moderate increasing, moderate decreasing, and the consistently low trajectories, respectively (*P* < 0.001).

While the moderate increasing and the moderate decreasing groups started at approximately the same level of adherence at baseline, they began to diverge at the 12-month visit. The results showed no significant differences in background characteristics between women in the two groups at baseline and visit number three as shown in Table 2.

### Adherence trajectories and time to virological failure

**Figure 3:**
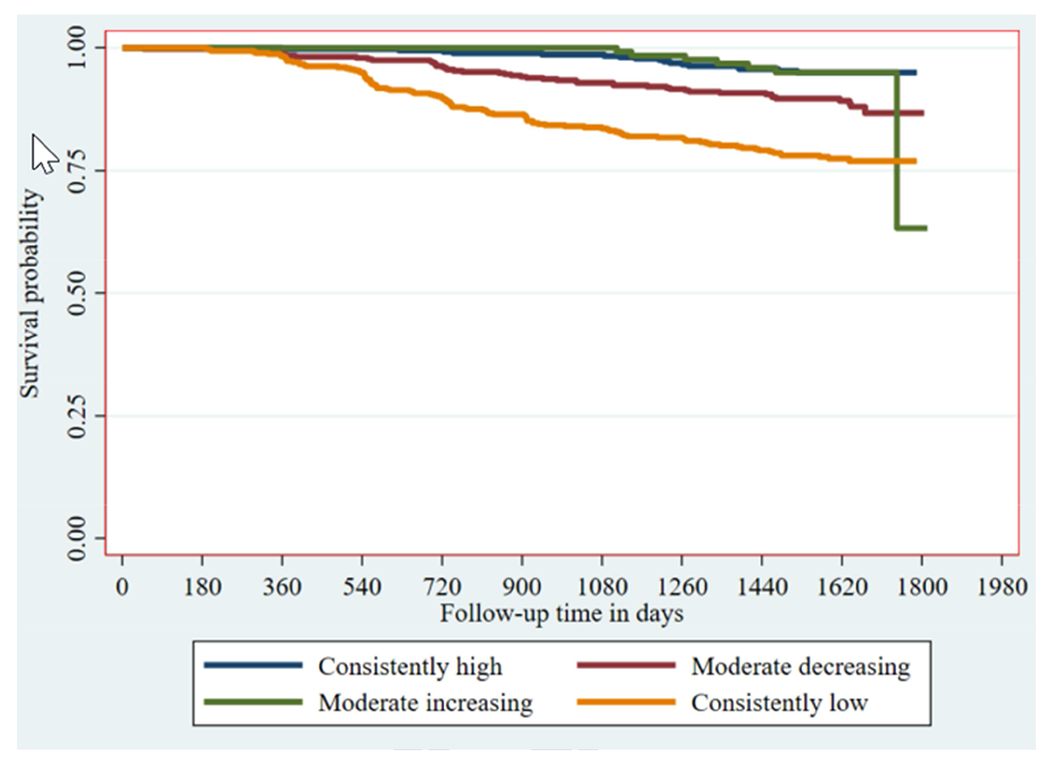
Kaplan Meier plot of time to virologic failure by adherence trajector

Virologic failure occurred among 173 of 1437 (12.0%) women. Women classified in the consistently low adherence trajectory experienced more virologic failure (103; 21.4%) compared to participants in the moderate decreasing (46; 10.2%), moderate increasing (7; 5.1%), and the consistently high (17; 4.5%) trajectories by the end of follow-up, (P <0.001). Figure 3 depicts time to virologic failure among the four adherence trajectories.

### Risk of virologic failure by adherence trajectories

Table 3 shows unadjusted and adjusted estimates of the risk of virologic failure by adherence trajectories from the Cox proportional hazards model. Compared to the consistently high trajectory, the adjusted hazard ratio (aHR) for the risk of virologic failure was highest among patients classified in the consistently low adherence trajectory (aHR 2.8; 95% CI: 1.6-4.9; P< 0.001) followed by the moderate decreasing group (aHR 1.8; 95% CI: 1.02-3.2; P=0.04). The risk of virologic failure in the moderate increasing was similar to the consistently high trajectory (aHR 1.0; 95% CI: 0.4-2.5; P<0.94).

## Discussion

Throughout the five-year study period, 12.0% of the women included in this analysis experienced virologic failure. GBTM revealed four latent trajectories of adherence to ART, with approximately one-third of women in the consistently low adherence group and one-quarter of the women classified in the consistently high group. The rest of the women were grouped into moderate decreasing and moderate increasing trajectories. The consistently low and moderate decreasing trajectories had higher hazard ratios of experiencing virologic failure compared to women who followed the consistently high and moderate increasing trajectories.

Our observed rate of virologic failure (12.0%) among women followed between 2014 and 2019 indicates a substantially lower rate of failure compared to a previous analysis of WIHS by McFall et al. [30] who found that nearly half of the women enrolled in WIHS between 2006 and 2011 experienced virologic failure. Notably, the adherence levels were similar between the two studies; 68% of the women reported at least a 95% level of adherence to ART in the study by McFall et al. compared to 67% of the women in the current analysis. The difference in the rate of virologic failure may therefore be attributed to the advancements made in the development of modern antiretroviral medications with low toxicities, coupled with the changes in the recommendations in 2012 regarding the initiation of ART irrespective of CD4 count compared to starting the treatment at prespecified cutoff points in the previous guidelines [31].

In this study, GBTM provided a novel insight into the dynamic nature of adherence behavior among WHIV in the US where we identified four categories of adherence over time. Previous studies have generally used the conventional approach of dichotomizing adherence or a maximum grouping into three levels of adherence [32]. In contrast to our findings, Storholm *et a*l [33] analyzed electronically monitored adherence using the Medication Event Monitoring System in approximately 240 Black PWHIV in the US at three points in time using GBTM. In that analysis, the model revealed three adherence trajectories, namely highly stable (40%), moderately low stable (35%), and low decreasing adherence (25%). The difference in the number of trajectories may be attributed to the difference in the sample size, the method of adherence measurement, and the time points at which adherence was measured. Interestingly, the percentages of the consistently high, consistently moderate, and consistently low adherence trajectories in our analysis supported the findings of the study conducted by Kassaye et al [4] who used GBTM to delineate the long-term HIV viral suppression trajectories among women in WIHS. In their analysis, the women were grouped into three trajectories based on the probability of viremia above 200 copies/mL as low (28.6 %), intermediate (39.4 %), and high (32.0%).

One potential advantage of GBTM in our analysis appeared in identifying latent moderate increasing and moderate decreasing groups; these trajectories started at similar levels of adherence but diverged at 12 months of follow-up. Our results showed no significant difference in the baseline characteristics at the start of the study or later at the time of divergence that would explain the observed change in the trajectory path of the two groups. However, given the complexity of the factors that affect adherence, qualitative in-depth studies are needed to explore the underlying reasons that led to the observed divergence [34]. Interestingly, the women who followed the moderate increasing trajectory compared to the moderate decreasing had an adjusted hazard ratio of virologic failure identical to those who were classified in the consistently high group. The experience of the women in the moderate increasing trajectory deserves further investigation to identify the factors that improved their adherence behavior; such data could be useful for developing adherence interventions for other women in similar circumstances.

The well-known relationship between adherence and viral suppression could be confounded by other factors with alternate, direct effects on viral suppression. In this analysis, we adjusted for such factors that were available in our dataset. Specifically, we controlled for the type of regimen at the last visit because of the variations in drug half-lives that influence the extent to which patterns of adherence results in viral suppression [23]. In addition, we adjusted for the frequency of experiencing viremia during the whole study period; the duration of prior viral suppression has a major effect on the level of adherence required to maintain suppression [24]. Furthermore, in the model, we accounted for the role of inflammation by controlling for the following variables: age, smoking, alcohol consumption, and depression [25,26,27], which could potentially impact the pharmacokinetics of ART and therefore viral suppression [35] [Supplemental Table 1]. We considered the many other individual, interpersonal, community, health system, and structural factors relevant to viral suppression; however, they all operate through adherence and were thus not added individually to the model [36].

Based on the results of this study, we identified several clinical recommendations that may improve nonadherence and decrease the rate of virologic failure. Firstly, future interventions should focus on women who follow low and moderate decreasing trajectories. Secondly, behavioral interventions to address the problem of heavy alcohol consumption among women who followed a consistently low trajectory are critical for increasing adherence and improving biological markers [37]. Lastly, among the same group of women, adherence can be improved by implementing cognitive behavioral therapy that concomitantly addresses both depression and poor adherence [38].

The major strengths of this analysis are the large sample size of participants who were followed for sufficient time to observe the outcome of interest. In addition, the data were nearly complete and there was only a slight difference between the number of viral load measurements and the number of self-reported adherence measurements. At the same time, we recognize that women who miss one or multiple visits may be exposed to social determinants of health that increase their risk of virologic failure. However, this analysis was not without limitations. Firstly, women self-reported their adherence to ART during the last month every six months, a method subject to recall bias and social desirability [39]. However, the self-report method can still provide useful information about adherence behavior as demonstrated in this analysis. In this respect, nearly 5% of the women classified in the consistently high trajectory experienced treatment failure, which may indicate misclassification bias in their adherence reporting or potentially use of suboptimal ART regimens (e.g., with drug resistance). In the future, objective measures of adherence like measuring the levels of ART in hair could provide less biased results about the association between adherence trajectories and treatment outcomes [40]. Secondly, we did not audit the prescribing practice of ART against the treatment guidelines to ensure conformity. Thirdly, even though WIHS recruited women from different geographical locations in the country, our result may not be generalizable to all WHIV. Future studies can utilize combined data from multiple databases. Lastly, we cannot exclude the impact of unmeasured residual confounding.

In conclusion, our study emphasizes that adherence to ART remains a challenge among HIV-positive women, as approximately one-third of the women were grouped into the consistently low adherence category. Grouping women based on their adherence to multiple trajectories rather than using the conventional method of dichotomizing them into adherent and nonadherent could be of great value to help in designing multilevel behavioral interventions that concomitantly address poor adherence, alcohol consumption, and depression, which are urgently needed.

## Supplementary Material

Supplementary material

## Figures and Tables

**Table 1.** Sociodemographic and clinical characteristics of women with HIV in the WIHS cohort by adherence trajectories

Characteristic	Adherence trajectories					
	Consistently high (n=378)	Moderate increasing (n=137)	Moderate decreasing (n=440)	Consistently low (n=482)	Total (n=1437)	P-value

Age groups						
<50 years	193 (51.0)	63 (46.0)	229 (52.0)	274 (56.8)	678 (47.2)	0.1
≥50 years	185 (49.0)	74 (54.0)	211 (48.0)	208 (43.2)	759 (52.8)	

Race						
African American	274 (72.5)	93 (67.9)	323 (73.4)	369 (76.6)	1059 (73.7)	0.19
Others	104 (27.5)	44 (32.1)	117 (26.6)	113 (23.4)	378 (26.3)	

Education						
Below secondary	141 (37.4)	48 (35.0)	134 (30.5)	146 (30.4)	469 (32.7)	0.03
Completed secondary	130 (34.5)	37 (27.0)	149 (33.9)	149 (31.0)	465 (32.4)	
Some college/completed college	106 (28.1)	52 (38.0)	157 (35.6)	185 (38.5)	500 (34.9)	

Employment status						
No	253 (66.9)	92 (68.1)	284 (64.8)	301 (62.6)	930 (64.9)	0.54
Yes	125 (33.1)	45 (32.9)	154 (35.2)	180 (37.4)	504 (35.2)	

Annual income						
≤$24,000	307 (81.2)	106 (77.4)	322 (73.2)	357 (74.2)	1,092 (76.0)	0.03
>$24,000	71 (18.8)	31 (22.6)	118 (26.8)	124 (25.8)	344 (24.0)	

Alcohol categories (%)						
0 drinks/week	249 (66.2)	88 (64.2)	242 (55.0)	212 (44.0)	791(55.1)	< 0.001
>0-7 drinks/week	97 (25.8)	43 (31.4)	158 (35.9)	194 (40.2)	492 (34.3)	
>7-12 drinks/week	15 (4.0)	2 (1.4)	15 (3.4)	29 (6.0)	61(4.2)	
>12 drinks/week	15 (4.0)	4 (2.9)	25 (5.7)	47 (9.8)	91(6.3)	

History of smoking status (%)						
Never smoker	140 (37.0)	48 (35.0)	147 (33.4)	168 (34.9)	503 (35.0)	0.49
Current smoker	125 (33.1)	46 (33.6)	176 (40.0)	183 (37.9)	530 (36.9)	
Former smoker	113 (29.9)	43 (31.4)	117 (26.6)	131 (27.2)	404 (28.1)	

Depression symptoms (%)						
Yes	77 (20.4)	39 (28.5)	130 (29.5)	192 (39.8)	999 (69.5)	< 0.001
No	301 (79.6)	98 (71.5)	310 (70.5)	290 (60.2)	438 (30.5)	

Regimen type at the last visit						
INSTI (without PI/NNRTI)	234 (61.9)	84 (61.3)	239 (54.3)	252 (52.3)	809 (56.3)	< 0.001
NNRTI (without PI)	72 (19.0)	30 (21.9)	84 (19.0)	71 (14.7)	257 (17.9)	
PI	65 (17.2)	19 (13.9))	89 (20.2)	93 (19.3)	266 (18.5)	
No therapy	1 (0.3)	0	18 (4.1)	54 (11.2)	73 (5.1)	
Others	6 (1.6)	4 (2.9)	10 (2.3)	12 (2.5)	32 (2.2)	

Episodes of detectable viremia						
Never	61 (16.1)	10 (7.3)	61 (13.9)	31 (6.4)	163 (11.3)	< 0.001
Infrequent	208 (55.0)	73 (53.3)	220 (50.0)	207 (42.9)	708 (49.3)	
Frequent	109 (28.8)	54 (39.4)	159 (36.1)	244 (50.6)	566 (39.4)	

Abbreviations: INSTI- Integrase Strand Transfer Inhibitor; NNRTI-Non-Nucleoside Reverse Transcriptase Inhibitor; PI- Protease Inhibitors

**Table 2.** Characteristics of women with HIV in moderate increasing and moderate decreasing adherence groups at baseline and visit three in the WIHS cohort

Baseline Visit					Third Study Visit			

Characteristic	Adherence trajectory		Total 577	P-value	Adherence trajectory		Total 577	P-value
	Moderate decreasing n (%) N= 440	Moderate increasing n (%) N= 137			Moderate decreasing n (%) N= 440	Moderate increasing n (%) N= 137		

Age groups								
< 50 years	229 (78.4)	63 (21.6)	292	0.21				
≥ 50 years	211 (74.0)	74 (26.0)	285					

Race								
African American	323 (77.6)	93 (22.3)	416	0.20				
Others	117 (72.7)	44 (27.3)	161					

Education								
Below secondary	134 (73.6)	48 (26.4)	182	0.30				
Completed secondary	149 (80.1)	37 (20)	186					
Some college/completed college	157 (75.1)	52 (24.9)	209					

Employment								
No	284 (75.5)	92 (24.5)	376	0.61	260 (75.4)	85 (24.7)	345	0.45
Yes	154 (77.4)	45 (22.6)	199		144 (78.3)	40 (21.7)	184	
Missing					36 (75.0)	12 (25.0)	48	

Annual income								
= < $ 24,000	322 (75.2)	106 (24.8)	428	0.32	298 (75.4)	79 (24.6)	395	0.25
>$ 24,000	118 (79.2)	31 (20.8)	149		102 (80.3)	25 (19.7)	127	
Missing					40 (72.7)	15 (27.3)	55	

Alcohol categories								
0 drinks/week	242 (73.3)	88 (26.7)	330	0.35	230 (73.3)	84 (26.7)	314	0.18
>0-7 drinks/week	158 (78.6)	43 (21.4)	201		139 (79.9)	35 (20.1)	174	
>7-12 drinks/week	15 (88.2)	2 (11.7)	17		12 (80.0)	3 (20.0)	15	
>12 drinks/week	25 (86.1)	4 (13.8)	29		22 (88.0)	3 (12.0)	25	
Missing	-				37 (75.5)	12 (24.5)	49	

History of smoking status								
Never smoker	147 (75.4)	48 (24.6)	195	0.35	134 (75.3)	44 (24.7)	178	0.83
Current smoker	176 (79.3)	46 (20.7)	222		154 (77.8)	44 (22.2)	198	
Former smoker	117 (73.1)	43 (26.9)	160		116 (75.8)	37 (24.2)	153	
Missing	-	-	-		36 (75.0)	12 (25.0)	48	

Depression symptoms								
No	310 (76.)	98 (24.0)	408	0.80	288 (75.6)	93 (24.4)	381	0.60
Yes	130 (76.9)	39 (23.1)	169		112 (77.8)	32 (22.2)	144	
Missing					40 (76.9)	12 (23.1)	52	

Substance use								
No	129 (74.6)	44 (25.4)	173	0.55	121 (74.2)	42 (25.8)	163	0.45
Yes	309 (76.9)	93 (23.1)	402		282 (77.3)	83 (22.8)	365	
Missing	2	-	2		37 (75.5)	12 (24.5)	49	

**Table 3.** Unadjusted and adjusted Cox Proportional Hazards Estimates of the Risk of Virologic Failure by Trajectories of Adherence

Covariates	Unadjusted HR (95% CI)	*P*-value	Adjusted HR (95% CI)	*P*-value

Adherence trajectories				
Consistently high	1.0		1.0	
Moderate declining	2.5 (1.4-4.3)	0.002	1.8 (1.02-3.2)	0.04
Moderate increasing	1.0 (0.5-2.7)	0.81	1.0 (0.4-2.5)	0.94
Consistently low	5.3 (3.2-8.9)	< 0.001	2.8 (1.6-4.9)	< 0.001
