# Supplementary material for: Trajectories of Antiretroviral Therapy Adherence and Virologic Failure in Women with HIV in the United States
Source: J Acquir Immune Defic Syndr. Author manuscript; Available in PMC 2024 Jun 1. (PMC10180014; doi:10.1097/QAI.0000000000003174)
Supplement: Supplementary material [file NIHMS1882904-supplement-Supplementary_material.pdf]

Supplemental Table 1

Table 3. Unadjusted Cox Proportional Hazards Estimates of the Risk of Viral Failure by Each Covariate of Interest

Covariates	Unadjusted HR (95% CI)	*P*-value
Age group		
≥ 50 years	1.0	
< 50 years	1.7 (1.3-2.4)	< 0.001
Alcohol categories		
Abstainer	1.0	
>0-7 drinks/week	0.9 (0.6-1.2)	0.46
>7-12 drinks/week	1.5 (0.8-2.9)	0.20
>12 drinks/week	1.6 (1.0-2.8)	0.05
History of smoking status		
Never smoker	1.0	
Current smoker	1.6 (1.1-2.3)	0.008
Former smoker	1.1 (0.7-1.6)	0.68
Depression		
No	1.0	
Yes	1.4 (1.1-2.0)	0.02
Regimen type at the last visit		
INSTI (without PI and NNRTI)	1.0	
NNRTI (without PI)	1.4 (0.9-2.4)	0.15
PI	3.4 (2.3-5.0)	< 0.001
No therapy	16.7 (11.3-24.9)	< 0.001
Others	0.5 (0.07-3.8)	0.52
Episodes of detectable viremia		
Infrequent	1.0	
Frequent	4.0 (2.8-5.5)	<0.001

Abbreviations: INSTI- Integrase Strand Transfer Inhibitor; NNRTI-Non-Nucleoside Reverse Transcriptase Inhibitor; PI- Protease Inhibitors
